# Classification Accuracy of Mixed Format Tests: A Bi-Factor Item Response Theory Approach

**DOI:** 10.3389/fpsyg.2016.00270

**Published:** 2016-02-29

**Authors:** Wei Wang, Fritz Drasgow, Liwen Liu

**Affiliations:** ^1^Department of Psychology, University of Central FloridaOrlando, FL, USA; ^2^Department of Psychology and School of Labor and Employment Relations, University of Illinois at Urbana-ChampaignChampaign, IL, USA; ^3^Department of Psychology, University of Illinois at Urbana-ChampaignChampaign, IL, USA; ^4^American Institutes for ResearchWashington, DC, USA

**Keywords:** mixed format test, bi-factor model, item response theory, constructed response items, classification accuracy

## Abstract

Mixed format tests (e.g., a test consisting of multiple-choice [MC] items and constructed response [CR] items) have become increasingly popular. However, the latent structure of item pools consisting of the two formats is still equivocal. Moreover, the implications of this latent structure are unclear: For example, do constructed response items tap reasoning skills that cannot be assessed with multiple choice items? This study explored the dimensionality of mixed format tests by applying bi-factor models to 10 tests of various subjects from the College Board's Advanced Placement (AP) Program and compared the accuracy of scores based on the bi-factor analysis with scores derived from a unidimensional analysis. More importantly, this study focused on a practical and important question—classification accuracy of the overall grade on a mixed format test. Our findings revealed that the degree of multidimensionality resulting from the mixed item format varied from subject to subject, depending on the disattenuated correlation between scores from MC and CR subtests. Moreover, remarkably small decrements in classification accuracy were found for the unidimensional analysis when the disattenuated correlations exceeded 0.90.

## Introduction

Large-scale testing has clearly moved from almost sole reliance on multiple-choice (MC) items in the mid to latter part of the twentieth century to the current use of mixed format tests that include both multiple-choice and constructed-response (CR) items (Ercikan et al., 1998; Kim et al., 2010; Kuechler and Simkin, 2010). For example, both MC and CR items are now employed in many tests including the National Assessment of Educational Progress (NAEP), the Advanced Placement Program (AP, College Board), SAT Reasoning Test (SAT, College Board), and Pre-Professional Skills Tests (PPST, Educational Testing Service). Lane (2005) found in a survey that 63% of state assessments have adopted a mixed format of MC and CR items and this number is increasing.

Many researchers believe that the combination of MC and CR items increases overall measurement accuracy, because the two item formats complement each other—CR items require more testing time but measure reasoning skills and in-depth knowledge that are difficult to assess with MC items. On the other hand, MC items are more efficient but some have argued that they only assess factoids of knowledge: They “yield a task that is abstracted too far from the domain of inference” (Wainer et al., 2001, p. 245). Moreover, MC items may be prone to test-wiseness contamination. Another advantage of CR items is that they may be able to provide information about students with extremely low or high abilities, which may be poorly assessed by MC items (Ercikan et al., 1998).

Although the mixed implementation of MC and CR items brings many psychometric advantages, it nevertheless leads to several important questions. First, perhaps one of the most fundamental questions regarding the mixed format test is whether the two item formats measure the same or highly similar constructs. This, in turn, leads to a further critical question for mixed format tests: Is it appropriate to use a unidimensional IRT model to simultaneously analyze data resulting from the two item formats? The application of unidimensional models to multidimensional sets of items has intrigued psychometricians for decades. Humphreys (1985) and Reckase (1979) argued that cognitive achievement tests are almost always multidimensional and Humphreys (1986) even further argued that minor dimensions should be deliberately included in a test in order to improve validity. Also, non-test related factors such as strategies for the speed of answering items, guessing strategies, and other test-wiseness strategies may also unintentionally create multidimensionality. In the context of the mixed format test, MC and CR items are sometimes designed and constructed for different testing purposes by different groups of test developers, thus they may naturally tap into different latent abilities.

In analyses of data from the College Board's Advanced Placement (AP) Program's mixed format Computer Science and Chemistry tests, Thissen et al. (1994) found some degree of multidimensionality. Ercikan and Schwarz (1995) found that two-factor models consistently fit mixed format test data better than one-factor models. This empirical evidence tends to suggest that it may be not appropriate to utilize unidimensional IRT models with mixed format tests. In fact, Ercikan et al. (1998) reported unidimensional calibrations of mixed format tests caused information loss for CR items.

Second, if an item pool consisting of MC and CR items is not truly unidimensional, then whether the unique ability associated with each item type can be accurately estimated becomes an important question. If the format specific factor cannot be estimated accurately, mixed format tests may actually omit something that subject matter experts and test developers believe to be important. Furthermore, identifying the unique abilities associated with each test format is important for subscore estimation. In the past years, although the testing industry has been increasingly interested in reporting subscores and diagnostic scores to either individuals or institutions (Haberman, 2008; Haberman et al., 2009; Haberman and Sinharay, 2010; Sinharay et al., 2010), obtaining reliable, valid, and meaningful subscores can be challenging, especially for mixed format tests. Moreover, IRT item parameters for the MC items and CR items are often estimated separately. We suspect that this approach may not be capable of providing accurate estimates of the reasoning skills uniquely assessed by CR items. Note that a high correlation between subscores computed separately from MC and CR items is expected because of the hierarchical structure of cognitive abilities (Carroll, 1993).

Lastly, important outcomes for many exams are based on a small number of score categories (e.g., pass/fail for licensing and credentialing exams, 1–5 for the College Board AP exams). To what extent does unidimensional calibration of mixed format tests undermine the classification accuracy of these high stakes scores? This is a practical yet very important question. Consider a student whose true standing on the constructs assessed by MC and CR items would lead to a composite AP score of 3, which would enable the student to receive college credit at many institutions of higher learning. What is the likelihood that such a student would receive a score of 2 (and not receive college credit) if the MC and CR items were calibrated together? Signal Detection Theory (SDT; DeCarlo, 2002, 2005) considers two types of misclassification: “misses,” or individuals who receive scores below the cut-off but whose true standing is above the cut-off, and “false alarms,” which consist of individuals who erroneously receive passing scores. Such misclassifications cause harm to students who are undercategorized (i.e., misses) and societal expense for students who are overcategorized (i.e., false alarms).

In the light of the discussion above, we propose a bi-factor model approach to analyzing and understanding the implications of the dimensionality of mixed format tests. With this model we can examine differences in classification accuracy of multidimensional and unidimensional calibrations.

The results of this study aim to contribute the literature on mixed format tests in several ways. First, the bi-factor framework provides a conceptual model for mixed format tests. Specifically, it assumes that an item measures a general ability common to all response formats and an ability uniquely assessed by the specific item format (i.e., MC or CR) that is orthogonal to the general ability (note also that the abilities uniquely assessed by item formats are orthogonal). To implement this model, we developed a computer program for ability estimation of the bi-factor model's general and unique abilities. Second, by comparing the classification accuracy of scores estimated for the bi-factor model with the accuracy provided by the traditional unidimensional approach, we advance understanding of the implications of the multidimensionality that is intrinsic to mixed format tests. Finally, by applying this method to analyze real data from 10 College Board AP Program tests with varying disattenuated correlations between the MC and CR subtests, we show the extent to which unidimensional calibrations lead to reduced classification accuracy.

### The bi-factor approach to item type analysis

**Figure 1:**
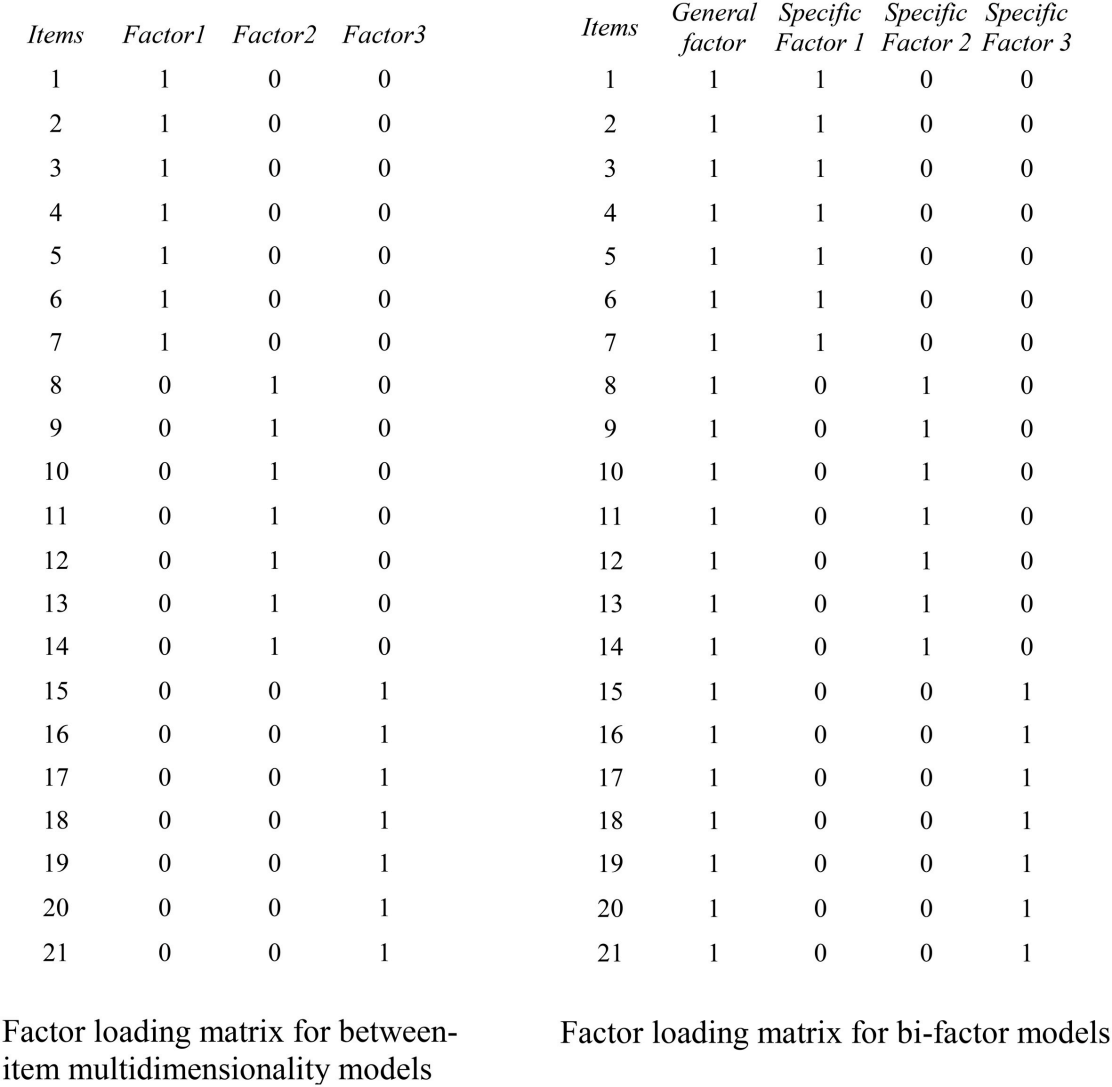
**Examples of factor loading matrices for between-item multidimensionality models (left) and bi-factor within-item multidimensionality models (right)**. The factors in between-item multidimensional models may be correlated whereas all factors in the bi-factor model are uncorrelated.

The bi-factor model has a long history (Holzinger and Swineford, 1937; Swineford, 1947, 1948, 1949), and it has recently enjoyed a strong resurgence of interest in psychometrics (Gibbons and Hedeker, 1992; DeMars, 2006; Reise et al., 2007; Gibbons et al., 2009; Rijmen, 2010; Cai et al., 2011; Jennrich and Bentler, 2011). Strikingly different from Thurstone's (1947) simple structure and the traditional between-item multidimensionality model—which assume each manifest variable measures only a single construct, and the constructs measured by different groups of manifest variables are correlated—within-item multidimensionality is assumed by bi-factor models: That is, each item is assumed to measure more than one latent construct (for a review of between-item and within-item multidimensionality models, see Adams et al., 1997). Specifically bi-factor models assume a *general factor*, which influences all items, and a number of *specific factors*, which affect different, mutually exclusive, groups of items. More importantly, bi-factor models assume all the specific factors are orthogonal with each other and with the general factor. Between-item multidimensional models and bi-factor models require different factor loading matrices as illustrated in Figure 1.

Recently, there have been important theoretical advances for the bi-factor model (e.g., Gibbons and Hedeker, 1992; Edwards, 2010a; Cai et al., 2011) and it has been applied to analyze the contributions of unique group factors in contexts such as the study of different facets of health problems (Reise et al., 2007), mood and anxiety symptoms in psychopathology research (Sims et al., 2008), and testlet-based assessment (DeMars, 2006). Reise et al. (2007) argued that a bi-factor model is a useful complement to traditional unidimensional analyses for three reasons: (1) it allows an examination of the distortions that may occur when unidimensional IRT models are fit to multidimensional data; (2) it enables researchers to empirically examine the utility of subscales; and (3) it provides an alternative to non-hierarchical multidimensional representations of individual differences.

Based on the previous research, we argue that bi-factor models are especially useful and appropriate for analyzing mixed format tests and examining the unique reasoning abilities and skills measured by CR items that cannot be measured by MC items. In our use of bi-factor models, we assume there is a single *general* knowledge and reasoning factor underlying performance on both the MC items and the CR items, and two test-format *specific* knowledge and reasoning factors, one for the MC items and one for the CR items, that are orthogonal to the general knowledge and reasoning factor.

These assumptions seem consistent with long-established theories and empirical findings. First of all, the idea of a general ability (vs. specific abilities) can be traced back to the seminal work by Spearman (1904, 1927) and is consistent with Carroll's (1993) three-stratum theory of intelligence. Soon after the debut of Spearman's theory of general intelligence, Holzinger, one of Spearman's PhD students, proposed a modified bi-factor model of intelligence (Holzinger and Swineford, 1937). The bi-factor model not only extracts the general factor (i.e., the *g* factor in Spearman's model) from all the measured variables, it also further analyzes the residual common factor variances into a number of uncorrelated group factors. The bi-factor model approach has been empirically found to be useful for intelligence measurement and research (Jensen and Weng, 1994). More practically, Gustafsson and Balke (1993) found that using both a general factor with a few specific factors together substantially improved the prediction of school achievement. Similarly, the bi-factor model appears to be a promising method for the analysis of mixed format tests as it allows simultaneous identification of general and specific traits.

The application of bi-factor models to mixed format tests is also consistent with the findings that CR items indeed measure unique abilities and reasoning skills that are different from MC items. CR items typically require responses ranging from short written answers to extensive essays or multiple-step solutions to complex problems. Thus, CR items are viewed as providing more information about certain deeper skills such as historical reasoning and the analysis of complex problems; they may also measure additional skills including reading and writing skills, even for mathematics tests (Ercikan et al., 1998). Behuniak et al. (1996) conducted a study using stem-equivalent mathematics items with CR vs. MC response formats and found that the CR-formatted items were more difficult than the MC-formatted items, although interestingly item discrimination was not significantly different across the two formats. Chan and Kennedy (2002) conducted a similar study with an economics test and also found that CR items were significantly more difficult than MC items for some questions. Thus, finding a psychometric model that adequately captures the unique reasoning skills associated with CR items becomes an important task for mixed format test researchers.

An important advantage of bi-factor models is that they facilitate the calculation of orthogonal subscores. As discussed above, the bi-factor model extracts the general factor and constrains the residual group factors to be orthogonal. The orthogonal nature of group factors in bi-factor models points to subscore estimation yielding scores that are mutually uncorrelated and uncorrelated with the general factor. This conceptualization of subscores is different from the traditional approach that sums the item scores from each format. The summed scores from each format are usually highly correlated, for they share the common variance of the general factor and may consequently provide little unique information. In contrast, the subscores estimated from the bi-factor model highlight the uniqueness of the group factors.

### Bi-factor model estimation

Although the bi-factor model appears to be a desirable approach to analyzing mixed format tests, its parameter estimation on the item level has been a challenge. The common approaches to estimation are structural equation modeling (SEM) and item response theory (IRT). Using traditional IRT based marginal maximum likelihood estimation with an EM algorithm leads to computations that are extremely demanding, especially when the number of factors is large. SEM with diagonally weighted least squares estimation for dichotomously or polytomously MC items also has a serious deficiency in that it is not full information. Gibbons and Hedeker (1992) made a fundamental contribution to the application of bi-factor models to item level data by discovering a way to compute marginal maximum likelihood estimates via a series of two-dimensional integrations regardless of the number of factors in a model. Gibbons and Hedeker's approach relied on a technique that limited its application to the normal ogive model. Recently, Edwards (2010a) developed a Markov chain Monte Carlo (MCMC) approach to the bi-factor item parameter estimation that he implemented (Edwards, 2010b) for a wide variety of models. Consequently, we used Edwards's (2010b) software.

MCMC is a very promising estimation method (Gilks et al., 1996). Although its full capacity for estimation has not been explored, several pioneering psychometricians have been amazed by the effectiveness of the MCMC estimation algorithm use with multidimensional estimation problems (see Shi and Lee, 1998; Béguin and Glas, 2001). For example, Bolt and Lall (2003) found that MCMC estimation is easy to implement, and that “algorithms for even complex multidimensional models can often be written in minutes” (p. 396). Edwards (2010a) has also asserted that MCMC will be an important estimation tool in the decades to come. A detailed introduction of MCMC estimation methods is beyond the scope of this paper. We direct interested readers to excellent sources such as *Markov Chain Monte Carlo in Practice* by Gilks et al. (1996), and *Monte Carlo Method in Bayesian Computation* by Chen et al. (2000).

### Dimensionality as a moderator for classification accuracy improvement

One of the goals of this study was to examine the improvement of classification accuracy resulting from the application of bi-factor models in comparison with the traditional unidimensional method, as classification decisions are often high stakes with very important consequences for test takers. We hypothesize that the degree of multidimensionality of the mixed format tests moderates the degree of improvement in the classification accuracy. Although we generally expected that the bi-factor approach to classification would outperform a unidimensional method, we specifically expected that the degree of improvement is greater for tests whose combined MC and CR item pool is substantially multidimensional than for tests whose MC and CR item pools are virtually unidimensional.

Previous research has found that the dimensionality of mixed format tests appears to vary across test subjects. For example, Bennett et al. (1991) examined the Computer Science test from the College Board's AP Program and found the test seemed to be unidimensional. In another study of the College Board's AP Chemistry test, Thissen et al. (1994) observed some degree of multidimensionality. Also, both Kuechler and Simkin (2004) and Bible et al. (2007) found a moderate relationship between students' performance on the two formats on the Information System examinations. In addition, Becker and Johnston (1999) reported that the Economics MC items and CR items were multidimensional, as they found that there was little relationship between the two test formats and concluded “these testing forms measure different dimensions of knowledge” (p. 348).

In this study, we use the disattenuated correlation between the summed scores for the two formats as an index of dimensionality. The College Entrance Examination Board (1988) reported varying observed correlations of mixed format tests across various subjects, with the correlation coefficients ranging from 0.47 for the Music Theory test to 0.73 for the Biology test and to 0.84 for the Chemistry test (also see Rosa et al., 2001). The correlations are expected to substantially improve for more recent test forms as their reliabilities have been improved in the past 20 years (Kim, 2010). We expected that the improvement of classification accuracy by using bi-factor models would be greater for mixed format tests with lower disattenuated correlations than for tests with higher disattenuated correlations.

## Methods

### Data

Data sets for this study were provided by the College Board. The tests analyzed included English Literature, World History, US History, European History, World History, and Physics B. For some of the tests, we analyzed data from two of the annual administrations (note that a different test form is administered every year). In total, data from 10 test forms were analyzed. The tests are listed in Table 1.

**Table 1:** **Classification accuracy of Unidimensional and Bi-factor approaches for 10 advanced placement tests**.

**Test**	**Year**	**Disattenuated Correlation^a^**	**NI^b^_CR_**	**NI^c^_MC_**	**Sample size**	**Accuracy by Unidimensional (%)**	**Accuracy by Bi-factor (%)**	**Accuracy improvement**
English Literature	2010	0.778	3	55	20,000	60.90	65.56	4.65%
English Literature	2009	0.77	3	55	20,000	63.04	67.17	4.13%
English Language	2009	0.81	3	55	20,000	63.62	67.56	3.94%
English Language	2010	0.807	3	54	20,000	63.37	67.15	3.78%
European History	2009	0.92	7	80	20,000	68.77	70.33	1.56%
World History	2009	0.89	3	70	20,000	69.70	70.75	1.05%
US History	2010	0.908	3	80	6936^d^	69.32	70.24	0.92%
European History	2008	0.89	7	80	20,000	68.14	69.06	0.92%
World History	2008	0.91	3	70	20,000	70.92	70.07	0.85%
Physics B	2008	0.96	7	70	11,941	76.73	77.06	0.33%

a *The disattenuated correlation denotes the estimated true score correlation between the subtest scores for the MC and CR items; they were provided by College Board*.

b *The number of CR items included in the tests*.

c *The number of MC items included in the tests. To maximize the comparability of results, we used 3 CR items and 55 MC items for all 10 tests*.

d *US History 2010 data set had a sample size of 20,000. However, only 6936 students chose the same three CR items (i.e., #1, #3, and #4 items)*.

We selected AP tests that varied in their disattenuated correlations between subscores from the MC and CR sections. The disattenuated correlations, computed as the observed correlation corrected by the classical test theory disattenuation formula, ranged from 0.77 for the 2009 English Literature test to 0.96 for the 2008 Physics B test^1^.

We obtained de-identified item responses of 20,000 test takers for each test from the College Board. However, we had to discard some data for the 2010 US History test and the 2008 Physics B test in order to maintain the same number of CR items for each test. The number of score categories of the CR items varied from 10 to 25 from test form to test form; we recoded them to a constant *C* = 5 score categories, *c* = 0, …, *C*−1, across all the tests by collapsing neighboring categories.

The number of CR items and MC items differed from test to test. In order to control the effect of number of items on classification accuracy, in our simulations we held the number of items constant for each format across all the tests. Specifically, the number of MC items simulated was controlled to be *N*_*MC*_ = 55 and the number of CR items simulated was controlled to be *N*_*CR*_ = 3.

### Estimation of item parameters and thetas

This study involved multiple steps of estimating item parameters and thetas. Using the original response data obtained from the College Board, we took two approaches to estimating the item parameters: We estimated the bi-factor model item parameters with MultiNorm (Edwards, 2010b) and we estimated unidimensional model parameters with MULTILOG.

MultiNorm fit the multidimensional 3-parameter normal ogive model (M3PNOM) to the MC items and the multidimensional graded response model (MGRM) to the CR items. These models were parameterized as:

(1) $$P\left(y_{j}=1|\underset{˜}{\theta }\right)=g_{j}+(1-g_{j})\Phi \left[{\underset{˜}{a}}_{j}^{\prime }\underset{˜}{\theta }-d_{j}\right]$$

and

(2) $$\begin{array}{l} P\left(y_{j}=c|\underset{˜}{\theta }\right)=\Phi \left[{\underset{˜}{a}}_{j}^{\prime }\underset{˜}{\theta }-\left(d_{j}+o_{jc}\right)\right]-\\ \;\Phi \left[{\underset{˜}{a}}_{j}^{\prime }\underset{˜}{\theta }-\left(d_{j}+o_{jc+1}\right)\right], \end{array}$$

where $\underset{˜}{\theta }$ is the vector of abilities [θ_*g*_,θ_*MC*_,θ_*CR*_], with θ_*g*_denoting the general ability, θ_*MC*_denoting the MC-specific ability, and θ_*CR*_denoting the CR-specific ability; ${\underset{˜}{a}}_{j}$ is the vector of discrimination parameters on the general factor and the specific factor for each item *j*, that is, ${\underset{˜}{a}}_{j}$ is ${\left[a_{g},a_{MC},0\right]}^{{}^{\prime }}$ for MC items and ${\left[a_{g},0,a_{CR}\right]}^{{}^{\prime }}$ for CR items; *d*_*j*_ is the item difficulty/location parameter for item *j*; *g*_*j*_ is the lower asymptote (i.e., guessing parameter) specific to MC items; *c* is the response category for the CR items, *c* = 0, 1, …, 4; and *o*_*jc*_is the category offset parameter for category *c* for item *j*. In this study, *o*_1_was conventionally constrained to be zero. To summarize, MultiNorm estimated four parameters for each MC item: *a*_*g*_, *a*_*MC*_,*d*_*MC*_, and *g*_*MC*_; and it estimated six parameters for each CR item: *a*_*g*_, *a*_*CR*_,*d*_*CR*_, *o*_2_, *o*_3_, and *o*_4_, because *o*_1_ = 0 and all the CR items were recoded to five response categories. The parameters for the two types of items were simultaneously estimated by MultiNorm.

With MultiNorm, we took an MCMC approach to the bi-factor item parameter estimation. Running MCMC was indeed time consuming and computationally intensive, mainly because we had a large number of item parameters to estimate (e.g., 362 item parameters to estimate for European History tests) with a quite large sample size (20,000). This took our lab computers about 5 s to run one cycle. As suggested by Edwards (2010b), we ran 60,000 cycles for each test and discarded the first 10,000 cycles as burn-in. 60,000 was substantially larger than many of the published MCMC applications, but this large number of cycles allowed us to discard a large burn-in and then extensively thin (thinning interval = 50) to avoid autocorrelation effects. After discarding the 10,000 burn-in cycles and drawing the remaining samples with a thinning interval of 50, we had 1000 draws left to estimate parameters and their standard errors.

We used MULTILOG (Thissen, 1991) to estimate simultaneously unidimensional model item parameters for the MC and CR items. In this approach, the MC items were parameterized by three-parameter logistic model (3-PLM; Birnbaum, 1968) and the CR items by the graded response model (GRM; Samejima, 1969). These models were parameterized as:

(3) $$P\left(y_{j}=1|\theta \right)=g_{j}+\frac{1-g_{j}}{1+e^{-a_{j}(\theta -b_{j})}}$$

and

(4) $$\begin{array}{l} P\left(y_{j}=c|\theta \right)=\frac{e^{-a_{j}(\theta -b_{jc})}}{1+e^{-a_{j}(\theta -b_{jc})}}-\\ \;\frac{e^{-a_{j}(\theta -b_{jc+1})}}{1+e^{-a_{j}(\theta -b_{jc+1})}}, \end{array}$$

where θ is the unidimensional ability; *a*_*j*_ denotes the discrimination parameter for MC and CR items; *b*_*j*_ and *g*_*j*_ are the difficulty and guessing parameters for MC items; and *b*_*jc*_ is the threshold parameter for category *c* for CR items. Therefore, in the unidimensional approach, MULTILOG simultaneously estimated three parameters for an MC item: *a*_*j*_, *b*_*j*_, and *g*_*j*_, and five parameters for a CR item: *a*_*j*_, *b*_*j*1_, *b*_*j*2_, *b*_*j*3_, and *b*_*j*4_.

### The simulation procedure

**Figure 2:**
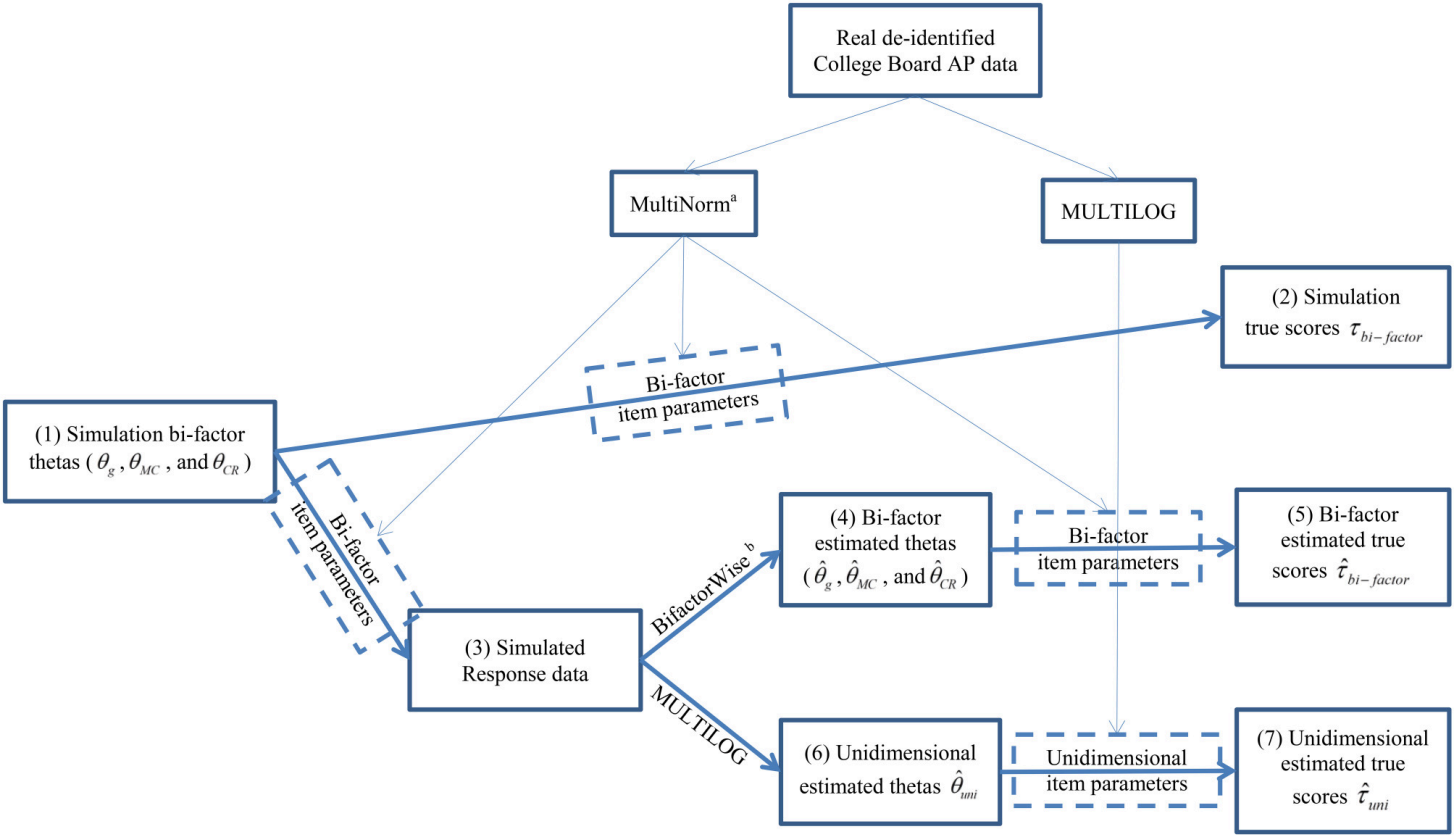
**Analysis procedures for this study**. The numbers in the parentheses in each box represents the procedural step described in Method section. ^*a*^The software MultiNorm was provided by Edwards (Version 1.0; Edwards, 2010b). ^*b*^The software BifactorWise was written by the authors and is available for free from the first author upon request. It estimates bi-factor thetas based on the response data and bi-factor item parameters.

Once the item parameters were estimated for the bi-factor and unidimensional models, we used simulation procedures to examine the accuracy of classifications. Comparing the classification accuracy from the two approaches provides an indication of the extent to which unidimensional modeling of multidimensional data leads to flawed decisions. Our analysis involved the calculation of three types of true scores: the simulation true score (τ_*bi*−*factor*_), the bi-factor estimated true score (${\hat{\tau }}_{bi-factor}$), and the unidimensional estimated true score (${\hat{\tau }}_{uni}$). These procedures included eight steps as detailed below and outlined in Figure 2. All the steps were implemented by a script written by the authors using the R statistic programming package (R Development Core Team, 2012).

The first step was to simulate bi-factor thetas for a sample size of 10,000 examinees. Following the assumptions of bi-factor models, the thetas for three latent traits (i.e., θ_*g*_,θ_*MC*_,θ_*CR*_) were assumed to be orthogonal; thus, the thetas for each simulee were independently sampled from a standard normal distribution. These simulated thetas, ${\underset{˜}{\theta }}_{bi-factor}=[\theta_{g},\theta_{MC},\theta_{CR}]\prime$ were treated as the true thetas and were used in the second step to calculate the simulation true score τ_*bi*−*factor*_ for each simulated examinee:

(5) $$\tau_{bi-factor}=1.125\times \tau_{MC,bi-factor}+2.75\times \tau_{CR,bi-factor}$$

where the constants 1.125 and 2.75 are weights used by the College Board to calculate the composite score for one of their AP exams, τ_*MC, bi*−*factor*_ and τ_*CR, bi*−*factor*_ are the true scores for MC items and CR items, respectively, calculated from:

(6) $$\tau_{MC,bi-factor}=\sum_{j=1}^{N_{MC}}P\left(y_{j}=1|{\underset{˜}{\theta }}_{bi-factor}\right)$$

and

(7) $$\tau_{CR,bi-factor}=\sum_{j=1}^{N_{CR}}\sum_{c=0}^{C-1}cP\left(y_{j}=c|{\underset{˜}{\theta }}_{bi-factor}\right),$$

where *N*_*MC*_ and *N*_*CR*_ are the number of MC items and CR items (*N*_*MC*_ = 55 and *N*_*CR*_ = 3 for all 10 studied tests); *C* is the number of score categories for the CR items (*C* = 5 for all CR items); and $P\left(y_{j}=1|{\underset{˜}{\theta }}_{bi-factor}\right)$, $P\left(y_{j}=c|{\underset{˜}{\theta }}_{bi-factor}\right)$ were calculated from Equations (1, 2).

The third step was to simulate response data based on the simulation bi-factor thetas and the estimated bi-factor parameters. We followed the conventional way to simulate response data: we calculated the probability of correctly answering an MC item and the cumulative probability vector (i.e., a vector [$P_{i0},\sum_{c=0}^{1}P_{ic},\sum_{c=0}^{2}P_{ic},\sum_{c=0}^{3}P_{ic},\sum_{c=0}^{4}P_{ic}$]) for a CR item by again using (Equations 1, 2), and then generated a random number from a uniform distribution *U*(0, 1). The response to an MC was determined by the comparison between the generated random number and the calculated probability, with 1 determined if the calculated probability was greater than the random number and 0 otherwise. The response to a CR item was determined by the location of the randomly generated number on the cumulative probability vector.

With the response data simulated, the fourth step was to estimate the bi-factor thetas by using the software BifactorWise^2^ written by the authors. This software adopted the maximum a posteriori (MAP) estimation method with the BFGS (Broyden-Fletcher-Goldfarb-Shanno) quasi-Newton estimation algorithm to estimate the three bi-factor thetas for each simulated examinee: θ_*g*_, θ_*MC*_, θ_*CR*_. These bi-factor estimated thetas, along with the bi-factor item parameters, were used in the fifth step to calculate the bi-factor estimated true score ${\hat{\tau }}_{bi-factor}$, by using (Equations 8–10):

(8) $${\hat{\tau }}_{bi-factor}=1.125\times {\hat{\tau }}_{MC,bi-factor}+2.75\times {\hat{\tau }}_{CR,bi-factor},$$

(9) $${\hat{\tau }}_{MC,bi-factor}=\sum_{j=1}^{N_{MC}}P\left(y_{j}=1|{\hat{\underset{˜}{\theta }}}_{bi-factor}\right),$$

and

(10) $${\hat{\tau }}_{CR,bi-factor}=\sum_{j=1}^{N_{CR}}\sum_{c=0}^{C-1}cP\left(y_{j}=c|{\hat{\underset{˜}{\theta }}}_{bi-factor}\right),$$

where ${\hat{\tau }}_{MC,bi-factor}$ and ${\hat{\tau }}_{CR,bi-factor}$ are the bi-factor estimated true scores for MC and CR items respectively; and ${\hat{\underset{˜}{\theta }}}_{bi-factor}$ is the vector of estimated bi-factor abilities [${\hat{\theta }}_{g}$,${\hat{\theta }}_{MC}$,${\hat{\theta }}_{CR}$] calculated from BifactorWise in the fourth step.

The sixth and seventh steps involved estimating the unidimensional thetas ${\hat{\theta }}_{uni}$ by running MULTILOG and calculating the unidimensional estimated true score ${\hat{\tau }}_{uni}$ by Equations (11–13):

(11) $${\hat{\tau }}_{uni}=1.125\times {\hat{\tau }}_{MC,uni}+2.75\times {\hat{\tau }}_{CR,uni},$$

(12) $${\hat{\tau }}_{MC,uni}=\sum_{j=1}^{N_{MC}}P\left(y_{j}=1|{\hat{\theta }}_{uni}\right),$$

and

(13) $${\hat{\tau }}_{CR,uni}=\sum_{j=1}^{N_{CR}}\sum_{c=0}^{C-1}cP\left(y_{j}=c|{\hat{\theta }}_{uni}\right),$$

where ${\hat{\tau }}_{MC,uni}$ and ${\hat{\tau }}_{CR,uni}$ are the unidimensional estimated true scores for MC and CR items respectively; and ${\hat{\theta }}_{uni}$ is the estimated unidimensional ability.

Once we calculated the three types of true scores (i.e., τ_*bi*−*factor*_, ${\hat{\tau }}_{bi-factor}$, and ${\hat{\tau }}_{uni}$), the last step (the eighth step) was to determine the classification accuracy of ${\hat{\tau }}_{bi-factor}$ and ${\hat{\tau }}_{uni}$ vis-à-vis τ_*bi*−*factor*_. To this end, we classified the 10,000 simulated students into five categories (because the AP Program provides scores from 1 to 5) on the basis of the three types of true scores. Specifically, for each type of true scores, we classified the simulees with scores in the highest 11% into category V, scores in the next highest 18.8% were placed into category IV, the next 22.8% received scores in category III, the next 25.8% were assigned to category II, and the lowest 21.6% were placed into category I (these are the actual classification percentages for classification for one of the AP exams). We then constructed two-way tables cross-classifying category scores from τ_*bi*−*factor*_ with ${\hat{\tau }}_{bi-factor}$ and ${\hat{\tau }}_{uni}$. For example, if the classification by ${\hat{\tau }}_{bi-factor}$ perfectly matched the classification by τ_*bi*−*factor*_, the classification accuracy of ${\hat{\tau }}_{bi-factor}$ would be 100%.

One of the primary goals of this study was to determine the accuracy improvement by using ${\hat{\tau }}_{bi-factor}$ than by using ${\hat{\tau }}_{uni}$. To precisely determine the improvement, we replicated the classification accuracy calculation for 20 times. In other words, we replicated procedure Steps 1–8 for 20 times for each studied test.

## Results

The results for the 10 studied tests are presented in Table 1, which displays information regarding the 10 AP tests and the corresponding classification accuracy results. The seventh and eighth columns present the classification accuracy results by the unidimensional and bi-factor approaches respectively. The ninth column—the rightmost—presents the improvement in classification accuracy by using bi-factor approach compared with using the unidimensional approach. The table presents results ordered by the magnitude of improvement.

As Table 1 clearly shows, the largest improvement resulting from the application of bi-factor models occurred for the literature and language tests: the 2010 and 2009 English Literature tests exhibited the largest improvement—4.65 and 4.13% respectively, followed by the 2009 and 2010 English Language tests, which showed improvements of 3.94 and 3.78%, respectively. The lowest improvement occurred for the 2008 Physics B test, which only improved by 0.33%. The 2008 World History test and European History test and the 2010 US History test also exhibited small improvements: 0.85, 0.92, and 0.92%, respectively.

Interestingly, the magnitude of the classification accuracy improvement resulting from modeling the multivariate structure of tests with both MC and CR items closely corresponds to the magnitude of the disattenuated correlation between the MC and CR subtest scores: the improvement magnitude is negatively associated with the disattenuated correlation. This finding supports our hypothesis proposed in the Introduction Section.

Another interesting point is that the classification accuracy of the unidimensional approach decreased with decreases in the disattenuated correlation. As shown in Table 1, the classification accuracy of the unidimensional approach was 76.73% for the 2008 Physics B test, whose disattenuated correlation between the MC and CR subscores was 0.96; however, the classification accuracy of the unidimensional approach dramatically decreased to 60.90% for the 2010 English Literature test, whose disattenuated correlation was 0.778. In contrast, although the classification accuracy of the bi-factor model also exhibited a similar pattern, it did not decrease as much as the unidimensional approach, which shows the advantage of the bi-factor model in the analysis of mixed format tests.

## Discussion

Psychometricans are often fond of MC items because they can be answered relatively quickly so that tests with many items can be administered in short periods of time, resulting in high reliability. On the other hand, test developers are often fond of CR items because this format lends itself to the assessment of reasoning skills that appear difficult or impossible to assess with MC items. For example, the 2006 United States History AP exam contained a CR item that provided test takers with letters written by women in 1776, 1839, and 1861, excerpts from essayists written in 1787, 1845, 1846, 1853, and 1861, and a table showing occupations of female wage earners in Massachusetts in 1837. The CR question asked, “Discuss the changing ideals of American womanhood between the American Revolution (1770s) and the outbreak of the Civil War” (College Board, 2007, p. 34). This question, designed to assess historical reasoning, contrasts starkly with MC items such as “The Supreme Court ruling in *Korematsu v. United States* upheld the constitutionality of …” (College Board, 2007, p. 28), which seems to assess a factoid of knowledge.

This study attempted to quantify the extent to which CR items tap reasoning skills above and beyond the general knowledge and reasoning skills that are common to MC and CR items. To this end, we carefully modeled data from 10 AP test administrations with bi-factor models and unidimensional models. We then simulated large samples of test takers with the estimated bi-factor model and scored the resulting response patterns with a bi-factor IRT approach and with a unidimensional IRT approach. These scores were transformed to the AP's one through five score reporting scale and then cross-classified with the AP score derived from the “true” (i.e., simulation) trait values. The reduction in score accuracy resulting from the unidimensional approximation provides a quantitative measure of the extent to which CR items assess reasoning skills above and beyond the general knowledge and reasoning skill common to CR and MC items.

Perhaps the most significant finding is that ignoring the unique reasoning skills tapped by CR items never decreased the accuracy of reported scores by as much as 5%. This is surprising given the apparently dramatic differences in the nature of MC and CR items (see, for example, the items described previously).

It is not surprising that the multidimensionality posed by mixed item formats varied from subject to subject and was strongly aligned with the disattenuated correlation between the MC and CR subtest scores. When the correlation coefficient was as high as 0.96 (for instance, the 2008 Physics B test), the mixed form test was virtually unidimensional and the application of bi-factor models could provide little gain in classification accuracy. However, when the estimated correlation between MC and CR items was 0.80 or lower, the multidimensional of the mixed format test appeared to be important and we expected the bi-factor approach to scoring would substantially increase accuracy, compared to unidimensional scoring. For perhaps the most multidimensional test, this improvement was 4.65%. Of course, the evaluation of whether this is a large or small effect is subjective. However, if we consider the great number of students taking AP exams each year, the consequences seem substantial. For example, if 300,000 students take the English Literature test, an improvement of 4.65% in classification accuracy means that an additional 13,950 students would receive correct scores (i.e., the scores they would have received if their skills had been measured without error). Therefore, we warn against simply applying unidimensional IRT to the mixed format test literature regardless of the latent structure of the assessment.

Note also that this study has limitations. For example, we analyzed tests consisting of exactly three CR items and 55 MC items across the various content domains. We expect that the classification improvement of the bi-factor approach would be larger if a mixed format test included more CR items (e.g., 5–10 CR items) and fewer MC items. Indeed, in the testing industry, there has been a strong advocacy for including more CR items and fewer MC items. In such cases, we suspect that the bi-factor approach would substantially outperform the unidimensional approach. This certainly provides an opportunity for further research.

On the other hand, many researchers have found that MC items are more efficient than CR items. For example, Lukhele et al. (1994) reported that the time an examinee used to answer one CR item corresponds to the time it takes to answer 16 MC items. Therefore, we believe a test developer should carefully consider the tradeoff of the two types of items and seek a balance that optimally utilizes the two test formats, which is of course another avenue for future research. We also suggest that test developers carefully balance the pros and cons of applying bi-factor models. Although the advantages are clear—a bi-factor model theoretically better suits the multidimensionality of most latent traits of assessment and helps improve classification accuracy, the disadvantages are striking as well, especially considering the complexity of computation and analysis.

Because of the orthogonal nature of traits estimated by bi-factor models, the bi-factor approach to mixed format test analysis is also expected to contribute to subscore estimation. Reporting subscores may not only provide useful information to examinees individually, but also offers valuable and meaningful feedback to institutions such as high schools. One challenge for subscore estimation is to find effective ways to estimate subscores and avoid the problem of providing highly correlated scores. Perhaps the bi-factor approach may be useful way in this regard.

In addition, the bi-factor model can also be useful outside of the educational/intelligence testing domains and has great potentials in applied psychometrics for non-cognitive measurement such as the assessment of personality, attitudes, vocational interests, well-being, etc. Indeed, the research of bi-factor models in such non-cognitive domains has burgeoning. For example, Chen et al. (2012) examined the validity of personality from a bi-factor model approach. Interestingly, their study revealed differential validity of the general and specific factor of the Extraversion personality. Similarly, Leue and Beauducel (2011) took a bi-factor model approach to reanalyzing the PANAS data and found different factors. These studies have highlighted the importance of applying bi-factor models to the personality research. We hope our study will shed light on such research in the future.

In sum, this study used the bi-factor model to characterize the latent structure of mixed format tests. MCMC estimation was used to fit this model to 10 tests administered in the College Board's AP Program. The accuracy of unidimensional and bi-factor IRT scoring was evaluated for these tests. When the disattenuated correlation between MC and CR subtest scores is large, there was virtually no gain resulting from multidimensional modeling. On the other hand, with disattenuated correlations in the neighborhood of 0.8, gains of 3.78–4.65% in classification accuracy were observed.

## Author contributions

WW, conducted the research and wrote the manuscript; FD, supervised the project; LL, cleaned and organized the data and assisted with initial data analysis.

## Funding

It was funded by College Board with number PO-9011173.

### Conflict of interest statement

The authors declare that the research was conducted in the absence of any commercial or financial relationships that could be construed as a potential conflict of interest.

## Footnotes

^1^The estimated true score correlations were provided by the College Board.

^2^This software is available for free from the first author upon request.
